# Neuromyelitis Optica in a Nepalese Man

**DOI:** 10.1155/2017/8596781

**Published:** 2017-08-08

**Authors:** Yogesh Subedi, Utsav Joshi, Sanjeeb Sudarshan Bhandari, Ashbina Pokharel, Ashbita Pokharel

**Affiliations:** Institute of Medicine, Tribhuvan University Teaching Hospital, Kathmandu, Nepal

## Abstract

**Background:**

Neuromyelitis optica is a severely disabling inflammatory disorder of the central nervous system of autoimmune etiology that mainly affects the optic nerves and spinal cord. Here, we present a case report detailing a patient with tingling and weakness of right upper and lower limbs who was neuromyelitis optica immunoglobulin G-positive.

**Case Presentation:**

A 46-year-old Nepalese man presented to the hospital with a history of tingling and weakness of right upper and lower limbs that developed over a period of two months. Clinical evaluation showed diminished power across all major muscle groups in the right upper and lower limbs. Magnetic resonance imaging of his cervical spine showed T1 iso- to hypointense signal and T2 hyperintense signal in central cervical spinal cord from first to sixth cervical level, probably suggestive of myelitis or demyelination. The patient was immediately started on intravenous methylprednisolone. The diagnosis of neuromyelitis optica was later confirmed with strongly positive neuromyelitis optica immunoglobulin G.

**Conclusion:**

In resource limited setting, in the absence of tests for neuromyelitis optica immunoglobulin G, treatment was started and the patient's condition started to get better. Hence, early initiation of aggressive immunosuppressive treatment is essential in such cases.

## 1. Introduction

Neuromyelitis optica (NMO) is a severe inflammatory disorder of the central nervous system (CNS) of autoimmune etiology that mainly affects the optic nerves and spinal cord [1]. NMO cases have been reported in all continents and races, although the disease prevalence has been found to be higher in areas with black, Asian, and Indian population [2, 3]. Diagnosis of NMO is made based on the presence of core clinical characteristics like optic neuritis, acute myelitis, area postrema syndrome, acute brainstem syndrome, symptomatic narcolepsy, or acute diencephalic clinical syndrome with neuromyelitis optica spectrum disorder- (NMOSD-) typical diencephalic magnetic resonance imaging (MRI) lesions and symptomatic cerebral syndrome with NMOSD-typical brain lesions with or without NMO Immunoglobulin G (NMO-IgG) [4].

There has always been a controversy regarding whether NMO is a subset of multiple sclerosis (MS) or is a distinct entity. However, clinical and laboratory evidences suggest NMO to be distinct from MS and that the pathogenesis of NMO is mainly due to humoral mechanisms [5, 6]. The target antigen for NMO-IgG is aquaporin-4 (AQP4) water channel. In one study conducted among Japanese patients, the sensitivity of anti-AQP4 antibody assay was 91% for NMO and 84% for high risk syndrome, and the specificity for both NMO and high risk syndrome was 100% [7].

No cases of NMO have been documented from Nepal and this may probably be the first case reported from Nepal. Due to poor economic status and resource limited settings in a country like Nepal, such disease is still underdiagnosed or misdiagnosed. Here, we present a case of a 46-year-old male with a history of sudden onset, painful diminution of vision and tingling and weakness of right upper and lower limbs, who was eventually diagnosed to have NMO.

## 2. Case Presentation

A 46-year-old Mongolian male, married, migrant worker presented to our department of neurology in the university hospital for the evaluation of tingling sensation and weakness of right upper and lower limbs.

Two months prior to his presentation to our hospital, the patient had nausea, hiccups, and epigastric pain. These symptoms were not accompanied by vomiting. Nausea and hiccups lasted for a week and they were not so severe as to require medical attention. A week later, he started to develop tingling sensation over his right upper limb. This persisted for nearly five weeks without the development of any other symptoms. Two weeks prior to the presentation, the tingling sensation progressed to involve the right lower limb. The tingling sensation was eventually followed by weakness in both right upper and lower limbs. The weakness in his right upper limb progressed to his right lower limb within a day and, thereafter, remained static. For these complaints, he was initially evaluated at a local hospital. He gave no history of weakness or sensory disturbances involving the left upper and lower limbs. There was no history of bowel or bladder incontinence. There was no history of fever, loss of consciousness, seizure, altered sensorium, or neck rigidity. However, he gave a history of sudden onset left eye pain followed by diminished vision which subsided on treatment two years back. There were no documentations available regarding the diagnosis and treatment for the same though. He did not have any history of alcohol intake or tobacco smoking. There was also no history of any chronic medical illness.

Clinically, his general condition was fair and stable. There was no pallor, icterus, cyanosis, oedema, palpable lymph nodes, or dehydration. On neurological evaluation, his higher mental functions and cranial nerves examination were intact. Eye evaluation with fundal examination was also normal at the time of presentation. Motor examination revealed normal muscle bulk but decreased muscle tone in both right upper and lower limbs. There was diminished power across all major muscle groups in the right upper and lower limbs. Power across the proximal muscle groups was 3/5 while it was 4/5 across the distal muscle groups in both upper and lower limbs. Deep tendon reflexes in both the limbs on the right side were moderately exaggerated (+++) whereas they were slightly exaggerated (++) on the left side. Plantar response was mute with diminished pin prick and fine touch sensation on the right upper and lower limbs while there was withdrawal plantar response with normal sensation on the left upper and lower limbs. Vibration and joint position senses were intact. Lhermitte's sign was absent in this patient. His gait, however, was unsteady.

Full blood count showed haemoglobin 14.1 g/dl, white blood cells 11,900/mm^3^ (neutrophils 76%, lymphocytes 22%, monocytes 1%, and basophils 1%), and platelets 152,000/mm^3^. His erythrocyte sedimentation rate (ESR) was 30 millimetres per hour. Biochemical evaluation showed random blood sugar level 9.7 mmol/l, urea 27 mg/dl, and creatinine 1.1 mg/dl. Urine routine and microscopic examination was normal. His vitamin B12 level was 764 pg/ml, which was within the normal range. His blood tests for human immunodeficiency virus (HIV), hepatitis B surface antigen (HBsAg), and hepatitis C virus (HCV) were negative. Venereal disease research laboratory (VDRL) test was also negative. Peripheral blood film for malarial parasite was negative. Serological evaluation for antinuclear antibodies and anti-double stranded-deoxyribonucleic acid antibodies were also negative.

**Figure 1:**
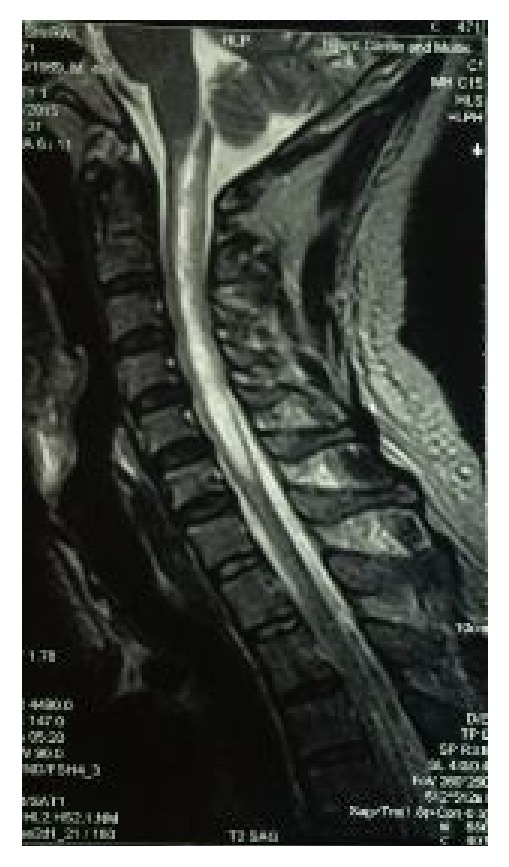
Magnetic resonance imaging of cervical spine; saggittal view of cervical segment of spinal cord is shown. The image shows T2 hyperintense signal in central cervical spinal cord from first to sixth cervical level, showing subtle enhancement and causing expansion of involved cord.

Nerve conduction study revealed normal amplitude, distal latency, and conduction velocity of right median nerve, right ulnar nerve, right common peroneal nerve, and right posterior tibial nerve. MRI of his cervical spine showed T1 iso- to hypointense signal and T2 hyperintense signal in central cervical spinal cord from first to sixth cervical level, showing subtle enhancement and causing expansion of involved cord (Figure 1). This was probably suggestive of myelitis or demyelination. Based on the clinical presentation and MRI findings, a provisional diagnosis of NMO was made and the patient was started on intravenous methylprednisolone 1 gram per day for three days and oral pregabalin tablets 75 mg twice a day for three days. Cerebrospinal fluid (CSF) analysis and antibody assay were not performed yet and the patient wanted to get referred to higher centre.

**Figure 2:**
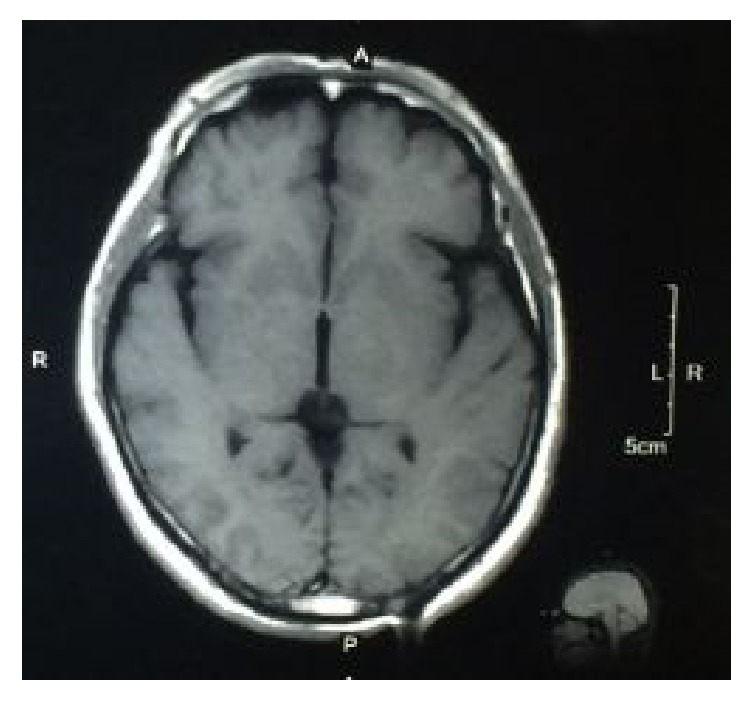
Magnetic resonance imaging of the brain; axial section of the brain is shown. The image shows normal study of the brain.

At our centre, lumbar puncture was performed which showed clear and colourless CSF, total count 25 cells/mm^3^ with all monomorphs, protein 15 mg/dl, and glucose 3.6 mmol/l. CSF adenosine deaminase (ADA) was within normal limit. Visual evoked potential (VEP) was also performed which indicated bilateral conduction defect. The P100 latencies were delayed for the right eye at both frequencies and for the left eye at 1 degree. The amplitudes were within normal limits for both eyes at both frequencies. Farnsworth Dichotomous Test for colour blindness indicated bilateral normal colour vision. MRI study of the brain was normal (Figure 2). NMO-IgG autoantibody test was still not available in our country at the time of presentation of the patient and, hence, the CSF sample was sent to India. Immunofluorescent assay revealed strongly positive NMO-IgG antibodies in the CSF sample. CSF immunoglobulin G (IgG) level was 52.7 mg/l which was also out of range (normal reference range: 0–34 mg/l).

Hence, the final diagnosis of NMO was made. Since he had already received a course of intravenous methylprednisolone for three days, he was given prednisolone 60 mg orally for a month and then in tapering dose. Oral pregabalin 75 mg twice a day was continued. His symptoms started to resolve and was discharged after a month of hospital stay. The patient and his family members were advised for physiotherapy for muscle strengthening exercises. He was able to walk with the help of a support during his discharge from the hospital. He was under regular follow-up for the next two months during which his muscle strength constantly improved. He was lost to follow-up after two months.

## 3. Discussion

This case report describes the complexities in diagnosing NMO in a resource limited setting.

NMO, a severe immune-mediated inflammatory demyelinating disease, is predominantly characterized by the combination of optic neuritis and myelitis [8]. Recent immunopathological studies have pointed towards aquaporin-4 as the target antigen in NMO and has been described to have a major role in the pathogenesis of NMO. Moreover, there is also a possible association of NMO with acute viral infection that may activate the immune system and initiate autoimmunity [9, 10]. NMO has been reported to occur in association with mumps, acute infectious mononucleosis, varicella zoster virus, pulmonary tuberculosis, and syphilis [9].

NMO is an inflammatory CNS disorder distinct from MS [4]. It is highly important to distinguish NMO from MS because the clinical course of NMO is more severe as compared to MS and the treatment is also different from MS [8]. Early-stage diagnostic specificity is essential as recent studies have suggested that interferon-*β*, natalizumab, and fingolimod may even worsen the course of NMO [4].

Optic neuritis and area postrema syndrome may precede the development of myelitis in a case of NMO. These clinical characteristics have been included as core features in the diagnostic criteria of NMOSD as well [4]. NMO may start in many cases with unilateral optic neuritis, which may be followed by the development of myelitis after a median of 14 months in AQP-4 IgG positive patients [11]. Intractable vomiting and hiccups may also herald the development of myelitis in such cases [12].

Even though the recent 2015 diagnostic criteria have clearly stated that the diagnosis of NMO can be made without AQP-4 IgG or even with the unknown AQP-4 IgG status, testing for NMO-IgG or AQP-4 antibodies has been largely helpful in laboratory diagnosis of NMO along with its differentiation from classical MS [4, 11]. The cell-based assays have been found to be most sensitive and specific for the detection of AQP-4 IgG antibodies in patients with NMO. However, immunohistochemistry or flow cytometry could be equally accurate in specialist centres [13].

In case of an acute initial presentation or exacerbation of NMO, the typical treatment is the administration of intravenous methylprednisolone therapy (IVMP; 1,000 mg daily for 3–5 days) and if there is no significant clinical improvement on steroids, plasma exchange (PLEX) has been shown to be effective for both optic neuritis and myelitis associated with NMO [14].

Based on prior history of unilateral optic neuritis and presence of contiguous spinal cord MRI lesion extending over ≥3 vertebral segments, the patient was immediately started on IV methylprednisolone. A high degree of suspicion is required to make the diagnosis this early in course of disease in the absence of NMO-IgG testing. In settings like ours, where serological assays may not be available, it is important to reckon clinical presentations and imaging findings so as not to delay the initiation of the treatment. After the patient was referred to higher centre on his own request, further investigations like brain MRI and NMO-IgG autoantibody test were performed that confirmed the diagnosis of NMO. Ideally he did not get a proper 5-day course of IV methylprednisolone but he was started on oral prednisolone as soon as the diagnosis was established. Hence, high degree of suspicion and proper judgement call in resource limited setting are always helpful to delay unfavourable outcome. In this case, he received proper medications timely.

## 4. Conclusion

Rapid diagnosis with early initiation of aggressive immunosuppressive treatment is essential in all the NMO cases. NMO is prevalent in our part of world as well but are not adequately diagnosed or misdiagnosed. So, availability of adequate imaging modalities and serological assays would be highly helpful to accurately diagnose NMO in our setting. If NMO-IgG autoantibody test can be made available within the country soon enough, this may help in prompt diagnosis and proper treatment.

## Data Access

The authors are happy to make readily reproducible materials described in the manuscript, including new software, databases, and all relevant raw data, freely available to any scientist wishing to use them, without breaching.
